# Emerging zoonotic diseases management: designing One Health interventions through a community lens including mental health

**DOI:** 10.3389/fpubh.2026.1900162

**Published:** 2026-10-09

**Authors:** Fatou Ndoye, Marjan Leneman, Jean Augustin Diégane Tine, Abdourahmane Sow, Elhadji Sow, Mouhamadou Wilane, Andree Prisca Ndour, Helena Ngowi, Gilbert Fokou, Benjamin Emikpe, Kathrin Heitz Tokpa, Bassirou Bonfoh

**Affiliations:** 1 VU Amsterdam, Social Sciences and Humanities, Organization Sciences, Amsterdam, Netherlands; 2 Faculty of Medicine, Pharmacy and Odontology, Institut de Sante et Development, Universite Cheikh Anta Diop de Dakar, Dakar, Senegal; 3 Department of Veterinary Medicine and Public Health, Sokoine University of Agriculture, Morogoro, Tanzania; 4 Diversity Focus-One Health Social Sciences (OHSS) and Food Systems, Nijmegen, Netherlands; 5 Institut Pasteur de Dakar, Dakar, Senegal; 6 Ministry of Livestock and Animal Production Service, Kédougou, Senegal; 7 Secretary of High National Council for Global Health Security Program, Dakar, Senegal; 8 Ecole Inter Etats des Sciences et Medecine Veterinaires de Dakar, Dakar, Senegal; 9 Centre Suisse de Recherches Scientifiques en Cote d'Ivoire, Abidjan, Côte d'Ivoire; 10 Dean School of Veterinary Medicine, Kwame Nkrumah University of Science and Technology, Kumasi, Ghana

**Keywords:** community based, global health security, governance, one Health, psychosocial effects, Senegal

## Abstract

**Introduction:**

The One Health (OH) approach has emerged as a comprehensive approach that recognizes the complexity of health challenges. Previous global health emergencies including Ebola and Covid-19 caused major social and psychological disruptions, generating fear, stigma, distrust, denial of disease and resistance to control measures. This study aims to investigate stakeholder OH perspectives and to assist in designing adaptations for interventions through a community lens, including for mental health.

**Methods:**

This mixed-methods study draws upon OH coordination activities and field experiences in Senegal and has been conducted between 2022 and 2024. Data sources include records of activities implemented through the National OH platform, epidemiological investigations carried out in collaboration with the Institut Pasteur de Dakar and the Ministry of Health and Social Action, and subsequent field investigations conducted in 2024. Qualitative information has been collected through participatory observation, field reports from eight OH coordination meetings, and nine semi-structured interviews conducted during the epidemiological investigations. In addition, 35 key informant interviews have been conducted with governmental, institutional, and community stakeholders involved in epidemic preparedness and response. Narrative accounts have been collected from 27 patients, survivors, and caregivers in relation to Covid-19, rabies, and Crimean Congo Hemorrhagic Fever (CCHF), as well as from four livestock keepers who had experienced animal losses. Quantitative data have been obtained through a survey of 434 livestock keepers assessing their knowledge, perceptions, and practices related to zoonotic diseases and OH interventions.

**Results:**

The study has identified several indicators of psychosocial distress among affected individuals and communities. Analysis of the OH coordination platform has shown that emergency responses were primarily organized around collaboration between the human, animal, and environmental health sectors, with operational activities mainly focused on surveillance, case detection, and contact tracing. Yet, social scientists, psychologists, and social workers were rarely involved in preparedness and outbreak responses. The study also revealed limited knowledge of zoonotic diseases among livestock keepers, with the majority (89.86%) recognizing only one or two diseases. Low knowledge was significantly associated with lower levels of formal education (*p* < 0.03). However, respondents demonstrated local knowledge of these diseases, describing some of them using local names. Most respondents (87.1%) reported no prior knowledge of the OH approach, while 75.81% were aware that diseases could be transmitted from animals to humans.

**Conclusion:**

The study suggests that OH interventions should be designed to bridge central decision making, operational planning, and community realities by integrating local knowledge, strengthening cross-sectoral collaboration and incorporating psychosocial dimensions into epidemic preparedness and response strategies.

## 1 Introduction

Emerging and re-emerging zoonotic diseases continue to threaten global health security, particularly in Africa, where recurrent viral disease outbreaks of Ebola, Marburg, Mpox, Crimean–Congo hemorrhagic fever (CCHF), and Covid-19 have exposed persistent weaknesses in epidemic preparedness and response systems (39). Beyond their often severe biomedical consequences, infectious disease outbreaks can generate widespread fear, social disruption, and economic instability, also exposing weaknesses in health systems and preparedness capacities (40). In response, the One Health (OH) approach has developed as a multisectoral framework that promotes collaboration across the human, animal, and environmental health sectors to strengthen disease prevention, surveillance, preparedness, and response (1, 2, 3, 40–42).

Following the 2013–2016 Ebola epidemic, several West African countries established national OH coordination mechanisms. In Senegal, the national OH platform, created in 2017 under the Permanent Secretariat of the High National Council for Global Health Security (SP-HCSS-OH), coordinates multisectoral collaboration and complements existing public health emergency systems, including the Public Health Emergency Operations Center (COUS) and the Integrated Disease Surveillance and Response (IDSR) system (4, 5, 43).

Although OH has substantially improved multisectoral coordination for zoonotic disease surveillance and response, implementation has largely focused on biomedical and institutional dimensions. Community engagement, psychosocial impacts, behavioral determinants, and mental health remain insufficiently integrated into OH preparedness strategies, particularly in low- and middle-income countries (6–8, 38). Previous epidemics, including Ebola virus disease and Covid-19, have demonstrated that fear, mistrust, stigma, and social disruption can undermine public health interventions and reduce the effectiveness of epidemic response (9, 40). The 2014–2016 West African Ebola epidemic exposed not only the fragility of health systems, but also the profound psychosocial consequences of outbreaks. Healthcare workers and affected communities experienced stigma, violence, grief, anxiety, depression, post-traumatic stress disorder (PTSD), and other forms of psychological distress, while mistrust of response measures and disruptions to cultural practices hindered disease control efforts (10, 11, 40, 44). Similar psychosocial impacts were observed during the Covid-19 pandemic, particularly in low- and middle-income countries, where fragile health systems, social isolation, economic hardship, and excessive workloads further exacerbated mental health burdens among communities and healthcare workers (12, 13, 45–47).

Women and girls have been disproportionately affected during epidemics, experiencing increased gender-based violence, educational disruption, child marriage, and other social vulnerabilities, particularly in settings with fragile humanitarian and health systems (14, 15). Similar patterns were reported during the Ebola outbreak in the Democratic Republic of Congo (DRC) (16). Regardless of growing recognition of these inequities, gendered and psychosocial dimensions remain insufficiently integrated into epidemic preparedness, surveillance, and emergency response strategies.

Despite growing adoption of the OH approach, its operationalization remains challenging in many African countries. Interdisciplinary collaboration, integration of social and behavioral sciences, and community engagement remain limited, while weak coordination, fragmented information systems, and insufficient adaptation of international frameworks to local contexts continue to constrain epidemic preparedness and response (3, 12, 40, 41). Joint External Evaluations of International Health Regulations (IHR) implementation has similarly identified persistent gaps in multisectoral coordination, surveillance integration, and emergency preparedness (17). In Senegal, although surveillance and OH coordination has improved, fragmented data systems and limited integration of community and psychosocial perspectives continue to constrain effective multisectoral preparedness and response (3, 43).

These gaps highlight the need for evidence on how community perspectives, psychosocial dimensions, and multisectoral coordination can be better integrated into operational OH systems. To address these gaps, this study investigates the implementation of the OH approach in Senegal by examining stakeholder perspectives across national, regional, district, and community levels. Specifically, the study (i) analyzes multisectoral coordination mechanisms for epidemic preparedness and response; (ii) explores community knowledge, perceptions, and lived experiences of zoonotic disease outbreaks; (iii) examines the psychosocial impacts of epidemics on affected populations and healthcare workers; and (iv) identifies context-specific interventions to strengthen community-centered and mental health-informed OH preparedness. The study is informed by systems thinking (18, 48) and socio-anthropological perspectives, which provide complementary frameworks for understanding interactions among institutional actors, communities, and health systems, as well as the social and behavioral factors influencing epidemic preparedness and response (19–21, 37).

## 2 Methods

This study employs an exploratory convergent mixed-methods design to investigate the implementation of the OH approach in Senegal, with particular attention to community perspectives and psychosocial dimensions of epidemic preparedness and response. It built on the following research questions: (1) What is the potential contribution of mental health in OH-related epidemic preparedness and response? (2) How can diverse stakeholder perspectives, including mental health actors, be aligned with community-based approaches in OH implementation? (3) How do community knowledge and perceptions of emerging health threats and OH shape intervention design? Qualitative and quantitative data and information were collected between 2022 and 2024 through participatory observation, epidemiological field investigations, key informant interviews (KIIs), case narratives, and a cross-sectional survey of livestock keepers. Data from these complementary sources were integrated during the interpretation phase to provide a comprehensive understanding of OH implementation across institutional and community levels.

### 2.1 Study area selection

The study was conducted in six of Senegal’s 14 administrative regions: Dakar, Saint-Louis, Kédougou, Kolda, Kaffrine, and Matam (Figure 1). These regions were purposively selected because they reported a high burden of zoonotic diseases according to the 2022–2023 epidemiological bulletins of the Ministry of Health and Social Action (MSAS), the Directorate of Livestock (DP), and the Ministry of Environment and Sustainable Development (MEDD). All selected regions had established permanent OH coordination committees and were regularly supported by the Directorate of Prevention/Public Health Emergency Operations Center (DP/COUS) during infectious disease outbreaks.

**Figure 1:**
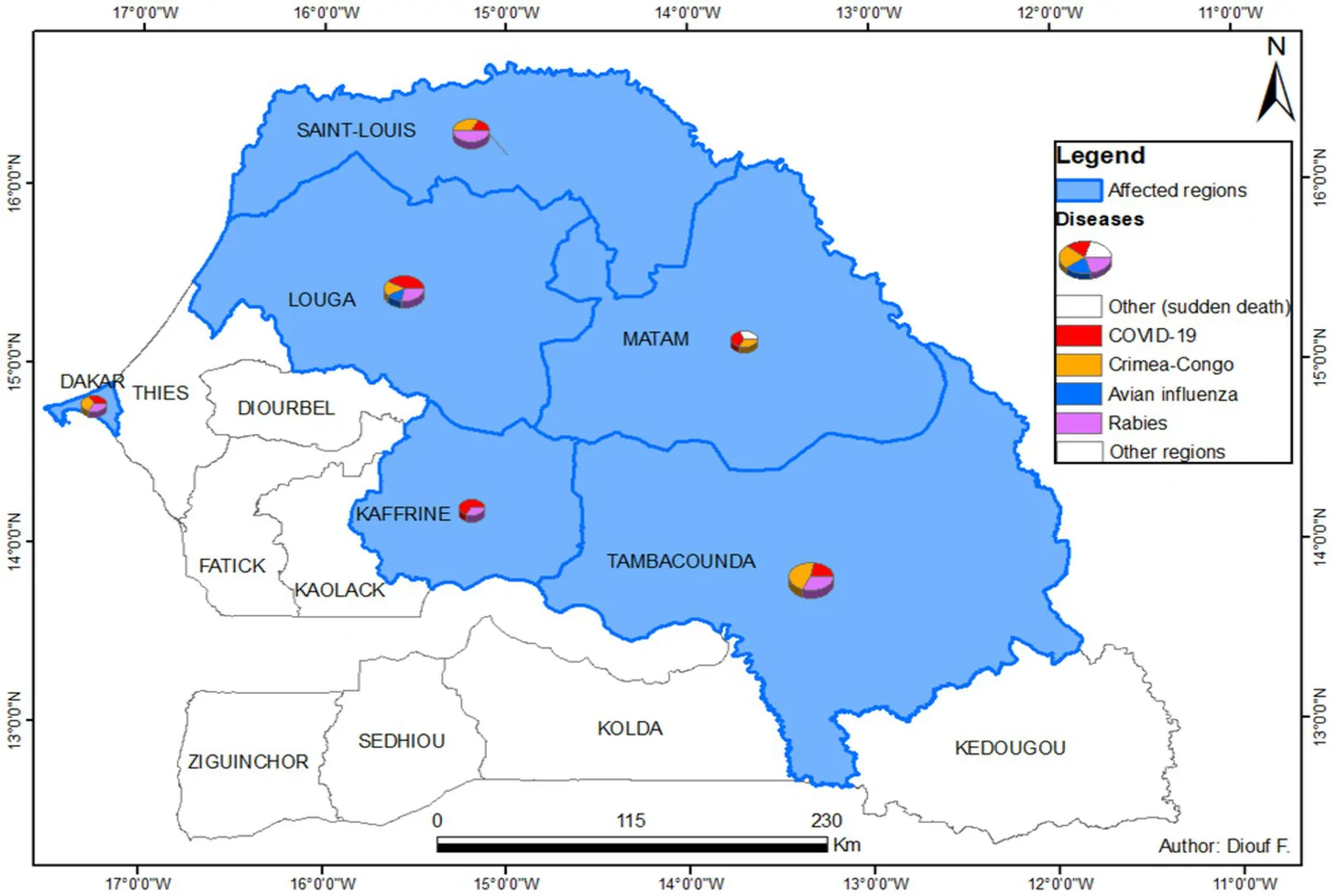
Study areas. Senegal Map 1 developed as part of the qualitative data collection conducted among victims in the targeted localities (2024), Public Health thesis, ISED/UCAD, Fatou Ndoye.

In total, qualitative data included field reports from eight OH coordination meetings, nine interviews conducted during epidemiological investigations, 35 semi-structured KIIs with governmental, institutional, and community stakeholders involved in epidemic preparedness and response, and 27 case narratives from patients and caregivers affected by Covid-19, rabies, and CCHF, together with four livestock keepers, who had experienced suspected zoonotic animal losses.

### 2.2 Ethical approval and qualitative study

The study received ethical approval from the National Health Research Ethics Committee of Senegal (CNERS). Oral informed consent was obtained from all participants prior to data collection, except for patients involved in epidemiological investigations, from whom written informed consent was obtained. The qualitative research component comprised four complementary data sources: (i) participatory observation during OH coordination meetings, (ii) interviews conducted during CCHF epidemiological investigations, (iii) KIIs and case narratives from affected individuals and livestock keepers.

Key Informant Interviews (KII’s) were purposively selected based on their direct involvement in epidemic surveillance, preparedness, and OH implementation. Selected key informants represented multiple sectors and administrative levels, including surveillance officers from the Directorate of Prevention/Public Health Emergency Operations Center (DP/COUS), the Directorate of National Parks, and the Directorate of Livestock Services at the national level. Selected key informants from district and community levels included District Chief Medical Officers (MCDs), Head Nurses of Health Posts (ICPs), assistant nurses, public and private veterinarians, para-veterinarians, veterinary auxiliaries, eco-guards, livestock keepers, butchers, community health workers (CVACs/community relays), social workers, and mental health professionals. This purposive sampling strategy ensured representation of stakeholders involved across the human, animal, and environmental health sectors.

#### 2.2.1 Participatory observation

Participatory observation, meaning that the researcher observes while participating in activities, was conducted throughout 2022 during regular meetings, multisectoral workshops, coordination sessions, and the annual steering committee meeting of the national OH platform. Detailed field notes were also kept on documented discussions, institutional interactions, coordination processes, and operational challenges. These observations provided contextual information on OH implementation and informed the development of subsequent interview guides for identifying key institutional issues requiring further exploration.

#### 2.2.2 Epidemiological investigations

During CCHF outbreaks in April and July 2023, field investigations were conducted in collaboration with the Institute Pasteur de Dakar (IPD) and Ministry of Health according to national epidemiological investigation protocols. As part of the multidisciplinary investigation teams supporting the Ministry of Health, socio-anthropologists documented the psychosocial dimensions of the outbreaks alongside routine epidemiological case investigations and contact tracing. Semi-structured interviews were conducted with confirmed patients, caregivers, and family members to explore disease perceptions, experiences with public health interventions, emotional responses (including fear and stigma), coping strategies, and psychosocial support needs. Participant observation further documented the implementation of OH interventions, the roles of different actors, and challenges encountered during case management and community follow-up.

#### 2.2.3 KII and case narratives

Between 2023 and 2024, additional In-depth interviews were conducted to explore multisectoral coordination, decision-making processes, and the integration of psychosocial support within epidemic preparedness and response. Interviews with patients, caregivers, and livestock keepers explored experiences of Covid-19, CCHF, rabies, and suspected zoonotic animal losses, including perceptions of illness, healthcare experiences, caregiving practices, stigma, fear, stress, and bereavement. Respondents were also asked about the social, financial, and psychological support received from families, communities, and institutions.

The Bereavement Risk Assessment Tool (BRAT) was used to guide the assessment of prolonged grief reactions, trauma-related symptoms, and persistent psychological distress among bereaved Semi-structured interviews with livestock keepers and other community actors explored their knowledge and perceptions of zoonotic diseases, preventive practices, and awareness of OH interventions. Interview narratives were analyzed to identify recurring themes related to bereavement experiences, disease awareness, and community responses.

### 2.3 Quantitative study

A cross-sectional survey was conducted among livestock keepers because of their frequent contact with domestic animals and their potential role in zoonotic disease transmission. A structured questionnaire was developed to assess knowledge and perceptions of zoonotic diseases and awareness of One Health interventions. The questionnaire was tested prior to data collection, and three trained peer researchers administered the survey infield face-to-face.

The sample size was calculated using Cochran’s formula (1977):

*n = Z^2^ × P × (1 − P) / E^2^* based on data from the 2023 National Population and Housing Census. According to the census, Senegal has 908,628 agricultural holdings, representing approximately 45% of all households (1,991,012) (33). Four regions with high concentrations of agricultural holdings - Dakar, Saint-Louis, Kédougou, and Kolda - were selected for the survey (Figure 2).

**Figure 2:**
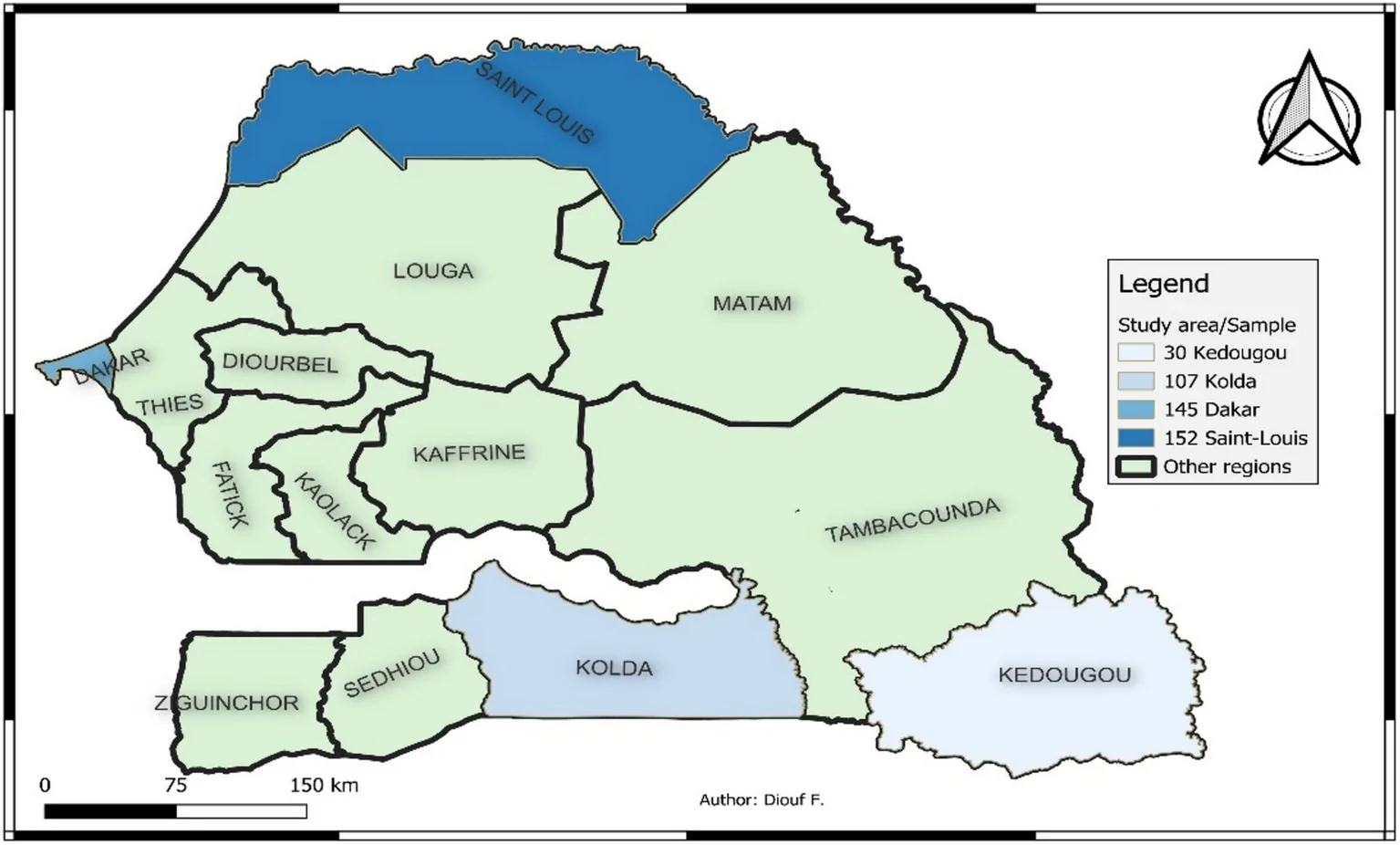
Study areas for the quantitative survey. Senegal Map 2 developed as part of the quantitative data collection conducted among livestock breeders in the targeted localities (2024), Public Health thesis, ISED/UCAD, Fatou Ndoye.

A total of 434 livestock keepers were selected using a cascade sampling approach. In the initial stap, communitiess were identified through local livestock networks and community leaders. Thesesubsequently facilitated access to eligible livestock keepers within their communities. Both men and women were eligible to participate. However, because livestock ownership is predominantly male in the selected rural communities, most respondents were men. Women identified as principal livestock owners were intentionally included in the qualitative component to better capture gender-specific perspectives (see Table 1).

**Table 1:** Overview of the mixed-methods study design.

Study phase	Data source	Participants	Purpose	Analysis
Phase 1 (2022)	Participant observation	OH platform members during 8 national coordination meetings	Explore OH governance, coordination processes and institutional dynamics	Thematic analysis
Phase 2 (2023)	Epidemiological investigations	9 interviews with confirmed CCHF cases, caregivers and family members	Explore psychosocial impacts, perceptions, stigma and implementation of OH during outbreak response	Thematic analysis
Phase 3 (2023–2024)	Key Informant Interviews (KIIs)	35 stakeholders from human, animal, environmental and social sectors	Examine OH implementation, multisectoral coordination and mental health integration	Thematic analysis
Phase 4 (2024)	Case narratives	27 patients/caregivers affected by COVID-19, rabies and CCHF, plus 4 livestock keepers experiencing animal losses	Document lived experiences, psychosocial impacts and community coping strategies	Narrative thematic analysis
Phase 5 (2024)	Cross-sectional survey	434 livestock keepers	Assess community knowledge and perceptions of zoonotic diseases and One Health	Descriptive and inferential statistics
Integration	Triangulation	All qualitative and quantitative datasets	Compare complementary findings and develop context-specific OH recommendations	Mixed-methods integration

### 2.4 Data management and analysis

Qualitative interviews were audio-recorded, transcribed verbatim, and imported into Atlas.ti 25 for thematic analysis. Coding followed an inductive-deductive method, based on the study objectives and emerging themes. The analysis explored stakeholder perspectives on OH implementation, multisectoral coordination, psychosocial impacts of epidemics, community knowledge and perceptions of zoonotic diseases, and the contribution of mental health and community health actors, including social workers, to epidemic preparedness and response.

Quantitative data were collected using KoBoCollect, exported to Microsoft Excel for cleaning, and analyzed using IBM SPSS Statistics. Descriptive statistics summarized participants’ socio-demographic characteristics and knowledge of zoonotic diseases. Associations between socio-demographic variables and knowledge levels were examined using chi-square tests and multivariable analyses. Participants identified diseases from a predefined list of 18 zoonoses, with one point assigned for each correct response. Knowledge scores were categorized as low (0–2 diseases), moderate (3–4 diseases), or high (≥5 diseases).

Qualitative and quantitative findings were integrated during the interpretation phase using triangulation validity. Evidence from participatory observation, interviews, case narratives, and the community survey was compared. This, to identify convergent and complementary findings regarding OH implementation, community perspectives, and the integration of psychosocial and mental health dimensions into epidemic preparedness and response.

## 3 Results

### 3.1 Mental health considerations in epidemic preparedness

The study has identified multiple indicators of psychological distress among affected individuals. The case narratives reveal emotional, cognitive, and behavioural responses to stressful or traumatic events, including illness, death, and livestock loss. Most interviewees? were either patients or primary caregivers experiencing both physical illness and emotional strain. Reported manifestations included fear, guilt, insomnia, intrusive memories, and symptoms consistent with post-traumatic stress. Psychological distress is particularly pronounced among adults aged 31–60 years, who often held central economic and caregiving responsibilities within households.

Psychological distress is associated with several contributing factors, including isolation during treatment, sudden bereavement, disrupted mourning practices, social stigma, and financial hardship. Stigma manifested through social avoidance, accusations of bringing curses, and calls to destroy personal belongings, further reinforcing social exclusion. Across diseases, caregivers often appeared more psychologically affected than patients, particularly during the Covid-19 pandemic and in cases of CCHF. Only two which ones, interviewees, or key stakeholders, livestock keepers, other? reported receiving professional psychosocial care, while most relied primarily on family support, which, although essential, remained insufficient. Formal mental health and psychosocial support (MHPSS) services were limited, inconsistently integrated into OH responses, and largely absent outside the Covid-19 context.

Interviews from the profiles of Covid-19 patients and healthcare workers confirmed that individuals with chronic conditions, as reported in 5 cases, such as diabetes, hypertension, cardiovascular diseases, or respiratory diseases, were among the most severely affected. The emotional burden was frequently compounded by financial hardship, particularly among low-income families facing major barriers to covering medication and hospitalization costs. This dual vulnerability - exposure to illness and economic strain - often worsened patients’ conditions and hindered recovery. A medical doctor acknowledged this: “*We observed a form of psychosis among the population, especially among patients with comorbidities, even before a diagnosis was confirmed*.”

During the Covid-19 pandemic, some *ad hoc* psychosocial support groups were established, such as psychosocial support units and call centers. However, these interventions were progressively interrupted as the crisis evolved, and no sustainable MHPSS mechanisms were maintained for subsequent outbreaks. Social workers reported that their roles remained largely confined to administrative functions, thereby limiting their contribution to epidemic preparedness and response despite the significant psychosocial distress observed during outbreaks “*So, for the Ministry, social workers are there to give discounts to patients who cannot afford it - this archaic reduction in healthcare coverage*” (Social worker). They also highlighted limited resources and insufficient recognition of their role as mental health and psychosocial professionals. “*We are not even consulted. I think that, to this day, people do not even know what the role of a social worker is”* (Social worker).

Social workers further reported having limited knowledge of diseases with epidemic or pandemic potential, such as hemorrhagic fevers, and little understanding of their psychosocial consequences, which constrained preparedness for addressing mental health needs during outbreaks. Albeit they consistently observed collective fear, psychosocial distress, and widespread misunderstanding within communities during the epidemics. Despite acknowledging the absence of formal psychiatric or psychological training, the social workers emphasized that they could substantially contribute to epidemic preparedness and response through patient accompaniment, community engagement, behavioral change activities, outreach, dissemination, and health education. They noted that such approaches had proven effective in other contexts, particularly HIV/AIDS programs, when social workers received appropriate training and resources. One social worker illustrated this during the Covid-19 pandemic. When patients failed to understand treatment procedures in isolation centers or disinfection measures upon discharge - generating fear, confusion, and psychosocial distress - social workers introduced preparatory counselling prior to hospitalization. According to respondents, frontline HCWs, such intervention facilitated the discharge process, reduced distress, and improved acceptance of care measures.

The absence of dedicated mental health professionals was also highlighted by healthcare staff: “*Here, there are neither psychologists nor psychiatrists. Illnesses are often considered normal and treated as such. Attention is focused solely on the disease.*” Psychiatric care was generally delivered in isolation by psychiatrists and primarily focused on severe or crisis cases, while broader psychosocial support, prevention strategies, and integration into epidemic preparedness remained underdeveloped. Frontline further emphasized the need to address psychological recovery alongside physical treatment, particularly during outbreaks: “*We need to treat the soul, not just the body*” (HCW). Although local epidemiological intervention teams investigated zoonotic outbreaks by collecting samples and providing advice, mental health support was not integrated into the response. Once intervention teams departed, no follow-up psychosocial support was provided. Families were left facing stigma, fear, and economic losses - all clear manifestations of psychosocial distress - while healthcare workers themselves struggled with exhaustion, hostility from communities, and insufficient training to address emotional and mental health needs. The absence of mental health professionals within OH coordination mechanisms appeared to weaken both preparedness and response capacities, leaving affected communities and frontline responders without adequate psychosocial support. “*During our interventions, we noticed that some people showed resistance, like the man who drove us out of his home when we tried to investigate; he probably had things on his mind that he did not want us to know. In general, it is the caregivers who are more mentally affected than the patients*” (ICP).

### 3.2 Differences in perspectives on OH and zoonotic diseases management

#### 3.2.1 Central level

At the central level, actors demonstrated a clear and comprehensive understanding of the OH approach, but they also highlighted several challenges that limit commitment among certain stakeholders. Respondents showed interest and expressed the need to collaborate and adopt the OH concept, citing examples from recent outbreaks. They noted that training sessions have already been delivered to MCDs, covering both OH and IHR. During a national OH coordination meeting, senior officials from health, livestock, and environmental ministries discussed integrating the OH approach into epidemic preparedness. However, these officials have reported uneven resource allocation across ministries, with each sector defining its own priorities and operating under constrained budgets. They highlighted the power asymmetries, particularly the dominant position of the Ministry of Health, which benefits from greater national budget allocations and more external support. This prioritization of human health was perceived to overshadow the interdependence with animal and environmental health as well as social and mental health sectors, leaving these sectors structurally under-resourced and insufficiently integrated into national planning.

Unclear who also spoke about a lack of trust between sectors. They explained that ministries are reluctant to share data, because they worry about how other sectors might use it, including any ethical issues involved. Their concerns especially relate to the possibility that shared information could be used for personal advantage or misused. When the discussion turns to joint surveillance protocols, each ministry insists on maintaining its own reporting channels and data systems (e.g., DHIS2 for human health, KoBoToolbox for livestock). According to, this reluctance to share information and harmonize systems makes it difficult to build an effective OH system. The national OH committee representative acknowledges that, while strategic coordination exists, operational actors are rarely involved in planning. As one officer puts it: “*We talk about One Health, but there is no solid coordination mechanism. In the end, ministries still work in silos, and each protects its mandate.*” (Sectoral officer 1).

OH is perceived as a necessary and intersectoral concept by central actors, but difficult to implement due to institutional fragmentation. Indeed, despite strategic coordination via the OH national platform with an existing OH committee and the presence of CNGE, practical implementation remains somewhat limited. Coordination meetings and workshops do occur, but mostly follow specific events or outbreaks. Inter-sectoral information exchange is mostly informal (e.g., email exchanges), and no real-time data-sharing platform is in place regarding epidemiological surveillance data. “*There is no real-time exchange platform. Strategic-level coordination exists, but the operational level is left out*.” (Sectoral officer 2).

Further, multisectoral meetings on zoonotic diseases surveillance were exclusively focused on medical and veterinary interventions, without discussions on mental health issues. Social workers and mental health professionals are absent from regional and central coordination, and they have reported a poor understanding of their role and lack of OH training. Senegal established the COUS, which includes an EMIS-Psy (Mobile Psychosocial Intervention Team) in 2017. Yet, both institutional analyses and field reports reveal several shortcomings in the implementation of this framework as not related to OH coordination. Interviews with practitioners further indicate that EMIS-Psy is not yet fully recognized by certain communities and segments of healthcare actors. Mental health professionals report that weaknesses in internal Mental health coordination undermine intersectoral collaboration and result in mental health being insufficiently prioritized within broader health activities. “*We have discussed this issue several times with the Ministry of Health, particularly the need to highlight psychological aspects. Meetings have been held on multiple occasions; however, once they return to their routine activities, these aspects are no longer considered a priority. This reflects the current reality*” (Mental health coordinator).

#### 3.2.2 Operational level

There is a large disparity in knowledge of the OH approach across actors. Some (such as MCDs) have received formal training, while others (e.g., auxiliary staff, para-veterinarians, social workers) are unaware of the concept or only vaguely familiar. As an auxiliary veterinarian reported: “*OH is more familiar at the departmental level. I never received training. As an auxiliary, I’ve just heard about it*.” Knowledge is often centralized at the departmental level, with rural actors feeling excluded. OH, remains unclear to many local health actors, both human and animal. “*I’ve heard of OH, but I do not know what it’s about; the concept seems interesting, but the population does not understand it very concretely*” (Health worker). “*I only hear about OH, but I do not know what it is. Perhaps this is because we do not have representation at the strategic level. Social workers at the hospital level remain confined to the hospital setting…*” (Social worker).

This lack of understanding is even more pronounced among social workers, who also reported a lack of awareness of public health policies and institutional frameworks. “*I am not familiar with the OH concept. We are not trained. We are not involved in activities or related initiatives. I understand the general idea of ‘health for all,’ but it is not discussed. We are in the field, in practice; we know nothing about health policy issues. We are in the hospital*.” (Social worker). According to this social worker, active during Covid-19, there is no knowledge of haemorrhagic fevers. *“We hardly participate in staff training activities. This is where the real problem lies. As far as I am concerned, and I do not know about others, I very rarely take part in training meetings. At the hospital, training sessions sometimes take place, but we are not involved. They are for medical and paramedical staff, but we are also part of the paramedical workforce. We clearly need continuous training, but unfortunately, we are not invited to training seminars. The social services department also does not organize training seminars. We are left on the sidelines.”*

Social services departments reported they are not involved in zoonotic disease cases, even though their mandates include psychosocial support. During the Covid-19 crisis, three active social workers voluntarily joined the medical teams. “*I did not wait to be called. I joined the doctors as soon as the response system was put in place*.” “*I said, listen, this is not only a medical issue; it is also a social one*.” (Social worker 1). “*I was at the heart of the response system because the professor understood the necessity of psychosocial intervention*…” (Social worker 2). However, institutional collaborations remained weak. “*Collaboration was lacking… There were sometimes serious tensions*” (Social worker 3).

Rural areas face a shortage of mental health professionals, with patients often referred to towns, but rarely attending. Health workers report minimal training, focusing on physical care while lacking skills to address emotional needs. “*One Health should also mean the health of the heart and mind*.” (HCW 1).

#### 3.2.3 OH coordination challenges during outbreak interventions

In each region, a human-health surveillance focal point reports routine cases from the district level to the central level (DP) for further investigation through MCD. When a suspected emergency disease occurs, the DP mobilizes the operational team. Surveillance actors report that relevant sectors (health, livestock, environment, hygiene services, park services, laboratory technicians, nurses, etc.) systematically convene when a case emerges, usually following a meeting with the MCD before action is taken. In epidemiological interventions, confirmation of a case immediately triggers contact or summoning of the patient to inform about the disease and thus informing on the urgency of further investigations. The alert is transmitted to the MCD and then to the DP, which deploys an operational team to implement and monitor interventions. Multisectoral actors then take necessary samples, inform families about the disease and required measures, and investigate contact cases.

However, field research highlights delay in the response to reported cases by operational actors. While the mechanism functions effectively at the local level, follow-up after investigations is often lacking. “*We send data to the Chief Medical Officer, who then sends it to the central level, but the experiences of families and patients are not transmitted*.” (Senior Head). According to social workers, at the central level there is an institutional interlocutor with the Minister of Social Action; however, this authority is not consistently present in the field. Social workers are required to compile data and transmit it to the central level, but they report important limitations in this process: “*We can transmit data, but there are things that cannot be transmitted - lived experiences…*.”

In the FHCC case, although sampling, confirmation of cases and contacts, and control measures was carried out, follow-up was insufficient. “*It’s pointless to conduct investigations, give advice, and leave without follow-up resources. The central level needs to react*.” (a District Officer). Delays are particularly evident at regional and central levels after reports and case data are submitted. The MCD confirmed coordination challenges and administrative slowdowns, with interventions sometimes occurring a week after deaths and recoveries. “*There are things that can only be resolved by being on-site.*” (HCW 2). Additionally, interventions are sometimes perceived as stigmatizing, generating rumours, fear, and concern within communities. In one region, a social worker’s assistant described involvement during an intervention: “*I went there with a medical team… we observed the patient, assessed his mental state… The presence of the medical team caused fear and panic among the relatives*….”

From field observations, epidemiologists involved are mostly public health officers with medical or veterinary backgrounds. Roles and responsibilities are not clearly defined, and interdisciplinary collaboration remains weak. HCWs pointed out coordination gaps during operations. In a rural district, a suspected rabies case was reported after a child was bitten by a stray dog. The nurse alerted authorities, but no veterinarian was dispatched. Rabies surveillance responsibilities remain unclear between sectors. Test results arrived 15 days later due to administrative delays. “*Since last year, there have been cases of dog bites, but rabies surveillance should be reconsidered.”* (HCW 3).

Medical professionals at the operational level acknowledge the link between human and animal health, but report insufficient training for collaboration with veterinarians. The private veterinary sector feels excluded. “*We are sometimes called only after the fact… the response is not integrated from the beginning public health services remain isolated in surveillance activities”* (a Veterinarian).

Field observations confirm that not all actors are involved and that the OH concept is not rigorously applied operationally. Roles and responsibilities are unclear, and socio-anthropologists and social workers are mostly excluded. Community actors report limited involvement in planning and decision-making, being engaged mainly during crises. Structural barriers - including poor mobile coverage, centralized health infrastructure, administrative delays, and overburdened staff - further limit participation. In remote areas, lack of incentives discourages sustained engagement, leading to reliance on donor-driven interventions that are not sustainable. Poor harmonization also leads to inefficiencies. “*Two vehicles are mobilized for the same investigation, each from a different ministry. We waste resources instead of coordinating*.” (MCD).

### 3.3 Community knowledge and perceptions on zoonotic diseases and OH awareness

#### 3.3.1 Socio-demographic characteristics of livestock keepers

Most of the respondents were male (74.65% out of 434; 95% CI = 70.6–78.7%), reflecting the local reality of a patriarchal social structure. The remaining 25.35% were female, primarily living in rural areas and belonging to the Fulani ethnic group (Figure 3).

**Figure 3:**
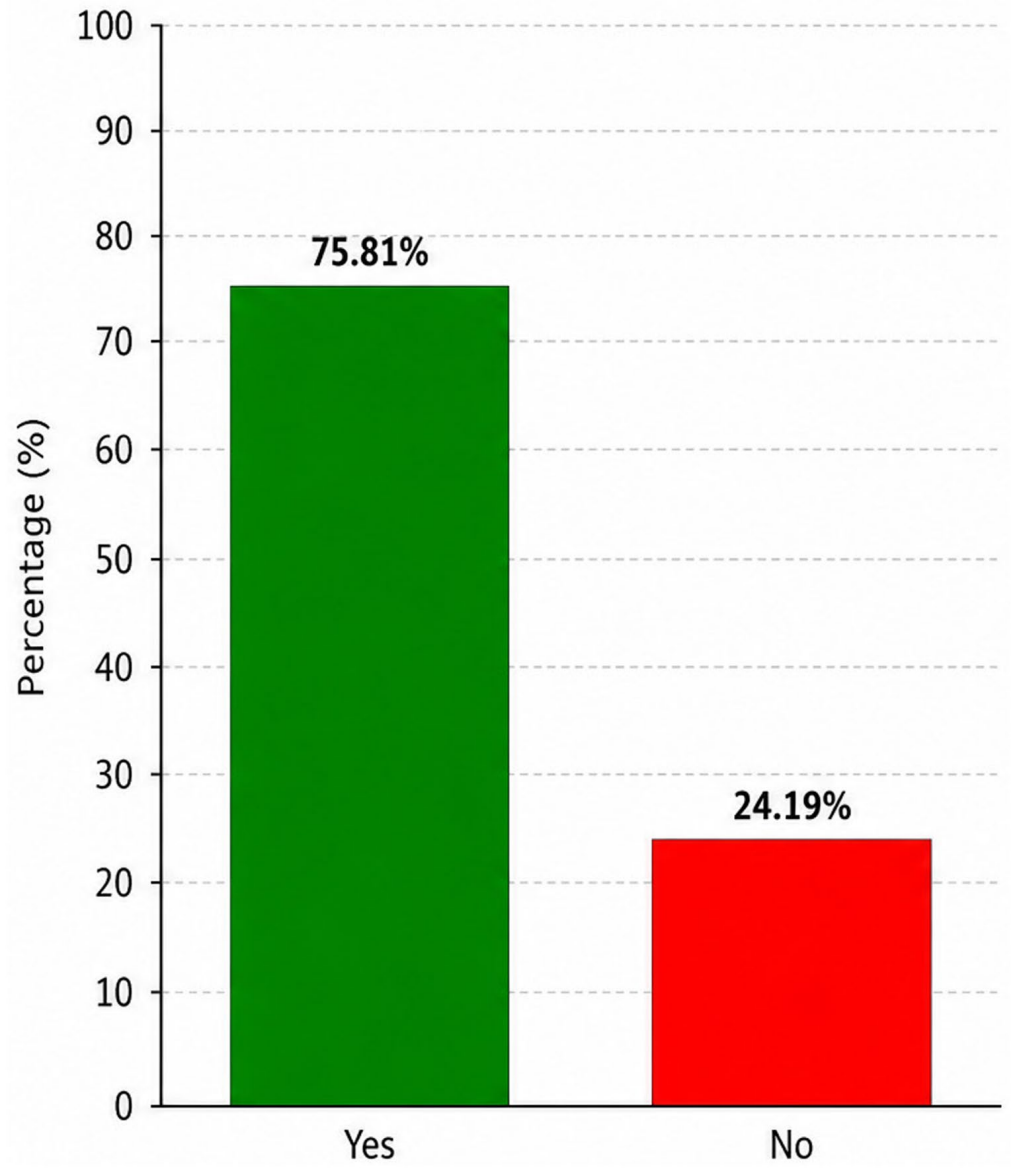
Awareness of animal-to-human disease transmission.

The respondents’ mean age is 45. The majority fall within the age ranges of 31–45 years (38.71%) and 46–60 years (40.32%) (Figure 4). Nearly 52% have no formal education, and only 14.06% have attained higher education. In terms of educational background, the majority received Daara (Koranic school) education (47.12%). Others received vocational or apprenticeship training (13.18%), Arabic or Franco-Arabic education (12.36%), or participated in literacy programs (1.65%). A small portion had no education at all (6.75%). Only 18.95% attended public or private French-language schools (Figures 5,6).

**Figure 4:**
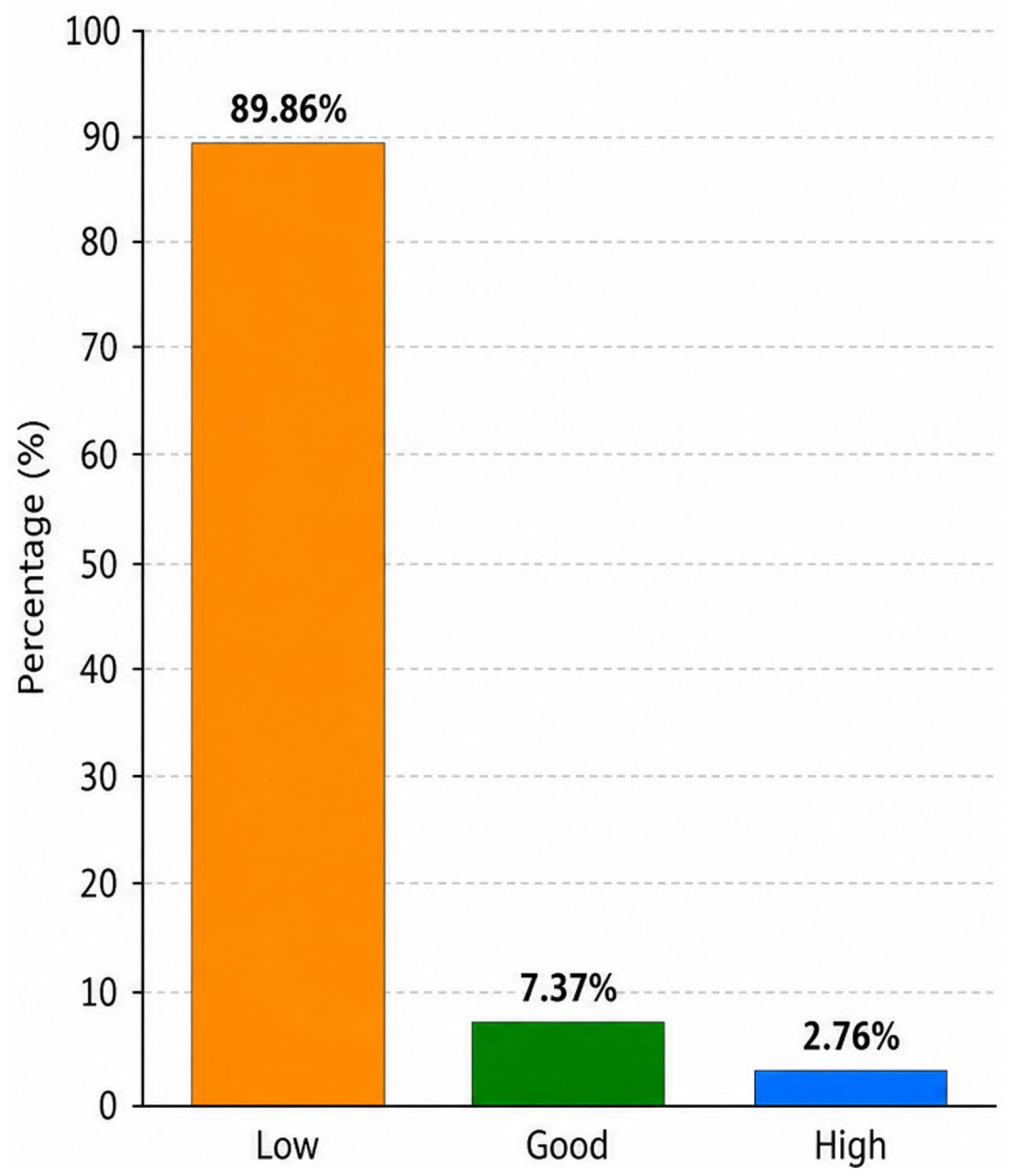
Self-reported knowledge level of zoonotic disease.

**Figure 5:**
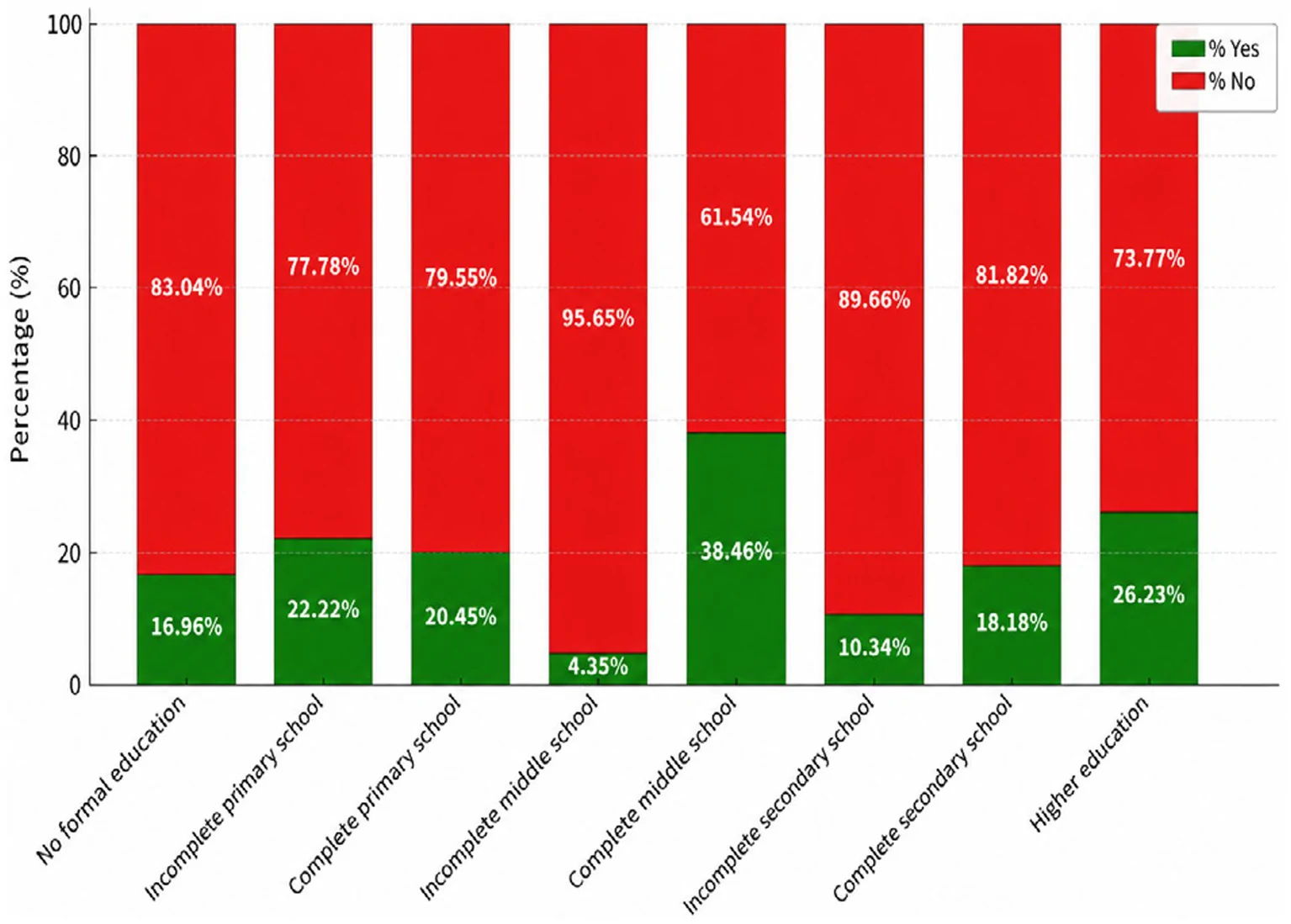
Knowledge of zoonotic disease by education level.

**Figure 6:**
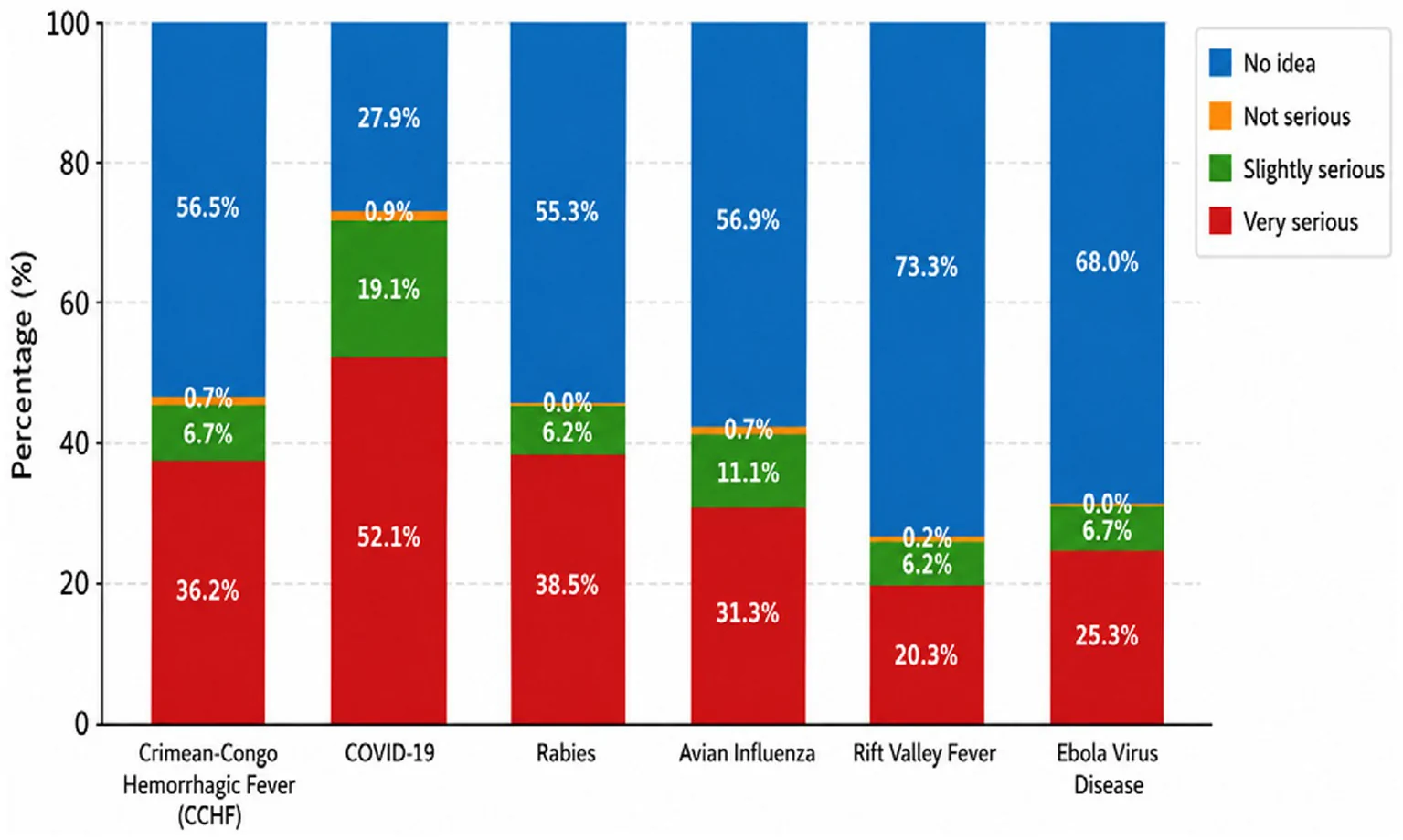
Perceived severity of zoonotic diseases (all categories).

The majority of the respondents (62.67%) reported that their spouses are also engaged in the same livestock activities. Given that most respondents are male, this indicates a significant level of involvement from female spouses in livestock work (Figure 7). For 58.06% of respondents, livestock is a secondary activity. In terms of income, 54.38% reported having occasional (seasonal) income, while 23.04% have no regular source of income. Only 17.74% receive a monthly salary, and 4.61% earn a daily income (Figure 8).

**Figure 7:**
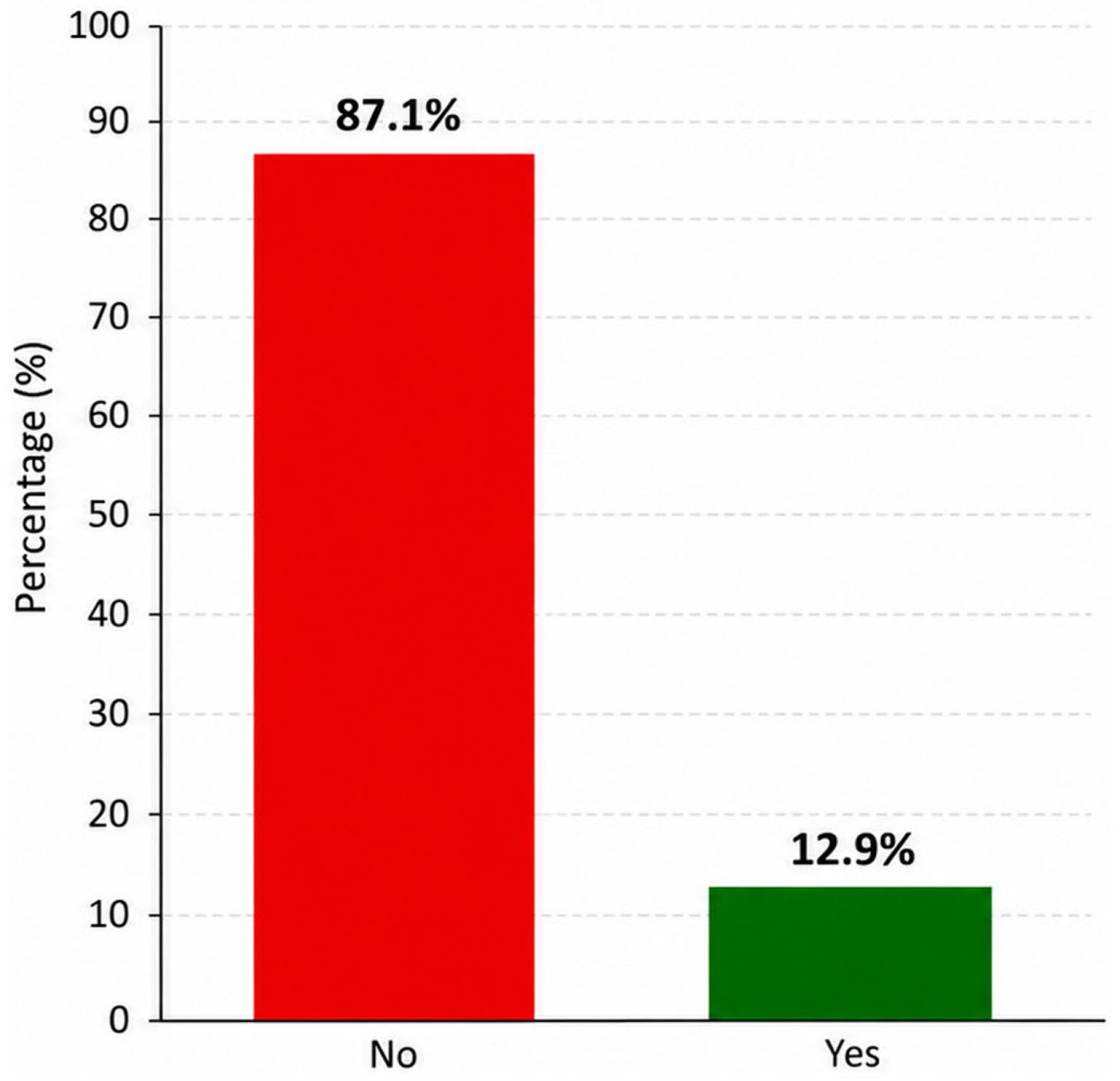
Awareness of the “One Health” approach.

**Figure 8:**
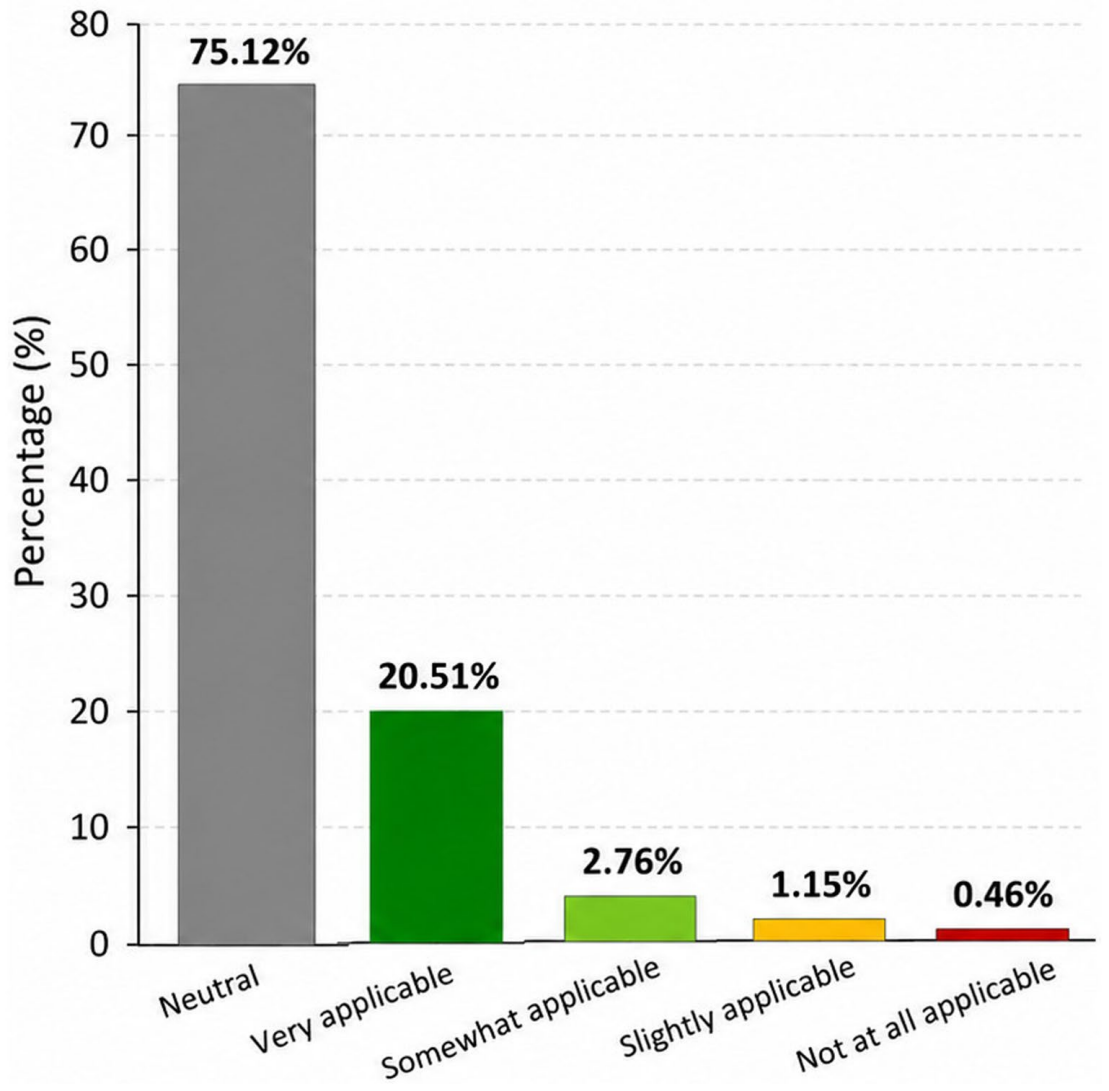
Perceptions of the applicability of the One Health approach.

#### 3.3.2 Knowledge assessment of zoonotic diseases among livestock keepers

Most participants (75.81%) are aware of diseases that can be transmitted from animals to humans (Figure 3). However, only an average of 52.07% are familiar with viral diseases transmitted by ticks to animals or humans, such as the emerging CCHF. After listing diseases individually to participants during the assessment, the majority (89.86%) demonstrated low knowledge, typically recognizing only one or two diseases. Only 7.37% showed good knowledge, and 2.76% demonstrated high knowledge (Figure 4). Qualitative data, however, revealed that interviewees possess local knowledge and provide explanations using local names or spellings for these diseases. The higher the level of education, the greater the likelihood of being aware of these diseases (*p* < 0.05). Men and women appear to have similar levels of knowledge based on the data provided (Figure 5).

The analysis highlights respondents’ knowledge regarding the perceived severity of different zoonotic diseases, as well as their understanding of these diseases within the local context (Figure 6). The findings reveal substantial variations in the level of knowledge depending on the disease considered. Regarding Covid-19, more than half of the respondents (52.1%) considered the disease to be very serious, while 19.1% perceived it as slightly serious and only 0.9% as not serious. However, 27.9% of respondents reported having no idea about the severity of the disease. These findings suggest that, despite the extensive media coverage and global impact of the pandemic, a considerable proportion of the population remains uncertain about its severity.

For CCHF, most respondents (56.5%) reported having no idea about the severity of the disease, whereas 36.2% considered it very serious. Only small proportions perceived it as slightly serious (6.7%) or not serious (0.7%). A similar pattern was observed for rabies, for which 55.3% of participants reported not knowing the severity of the disease, while 38.5% considered it very serious. Concerning avian influenza, more than half of the respondents (56.9%) also indicated that they did not know how to assess its severity. Nevertheless, 31.3% considered it very serious, whereas 11.1% perceived it as slightly serious. Knowledge gaps were even more pronounced for Rift Valley fever, with 73.3% of respondents reporting no knowledge of its severity and only 20.3% considering it very serious. A similar situation was observed for Ebola virus disease, where 68.0% of respondents reported not knowing its severity, while 25.3% considered it very serious (see Figures 7, 8).

Most respondents (87.1%) reported being unaware of the implementation of the One Health approach in their localities, although the Division of Veterinary Services has performed sensitization among the livestock keepers. Consequently, most responses (75.1%) remained neutral when asked about the perceived applicability of the approach in their area. The community at the household level did not make the link between transmission from humans to animals.

The qualitative data show that community actors recognize the OH aspect with multiple actors working together. As one community actor explained: “*Multi-actor means that each sector has its community actors, but the types of community actors differ, as do their missions and their understanding. In reality, they are just community actors more involved in advocacy, not professionals*.” The OH concept remains largely unfamiliar and unclear, with limited translation into practical action. “*I’ve heard about OH, but I do not know what it is*” (Livestock keeper). The concept remains vague and poorly adapted. “*It will be necessary to use many terms adapted to the community. For example, if you take the Lebou area* (an ethnic group with a fishing culture and traditional therapeutic rituals for mental health, Ndoep), *you can take an organization and find a message code adapted for co-construction with these communities. It will need to be conceptualized in relation to each community with the involvement of each actor* (i.e., the fishermen’s association, the *Ndeupeukat (therapeute of Ndep ritual)*.).” (community health leader).

The OH translations given in Wolof are “*Guir wér-gi-yaram bu mucc ayib*” (For complete/optimal health) or “*Ngir kaaraange askan wi*.” (For the safety/protection of the population). According to one community health actor, these terms are inadequate and may even create confusion, explaining that translating “*OH*” as “*benna weer gou yaram*” (in Wolof) does not reflect the intended meaning of OH. He recommends conducting community-level research to first explain the concept and objectives of OH, and then identify the most appropriate terms in local languages. As community actor stated: “*We should consult leading Wolof-language researchers to determine an appropriate term for ‘One Health*.”

Livestock keepers demonstrated extensive indigenous knowledge of animal health, using local disease names accompanied by detailed descriptions of clinical signs and traditional management practices. Several locally recognized disease terms, such as *Daasso* (associated with trypanosomiasis) and *Mbana* (a severe throat condition), were consistently described by respondents. However, the correspondence between local disease classifications and biomedical diagnoses was not always straightforward, highlighting potential discrepancies in disease terminology and the need for improved communication between livestock keepers and veterinary services.

Traditional animal healthcare practices remain widespread, particularly among Fulani pastoralists. Respondents described the use of salt, plant-based preparations, natural oils, and other locally available remedies to manage animal diseases before seeking veterinary assistance. These practices were considered effective by livestock keepers and were often preferred when veterinary services were inaccessible or unavailable.

Despite their extensive knowledge of livestock diseases, awareness of zoonotic diseases was generally limited. Rabies (*Say* in Pulaar) was widely recognized and perceived as a serious disease affecting both animals and humans. In contrast, awareness of priority zoonoses such as CCHF, Rift Valley fever (RVF), and West Nile virus was minimal among livestock keepers, butchers, and other community members. Respondents reported relying primarily on visual inspection of animals and meat and had received little or no formal training on zoonotic disease recognition or prevention.

Veterinarians reported diagnosing cases of CCHF, RVF, and West Nile virus in the study area, whereas community members generally became aware of these diseases only after livestock deaths or during disease outbreaks. This discrepancy illustrates a substantial gap between professional veterinary knowledge and community understanding of zoonotic diseases. Language and communication barriers further constrained zoonotic disease awareness. Health education activities were primarily delivered in Wolof, whereas many Fulani pastoralists had limited proficiency in this language. In addition, technical disease names such as CCHF and RVF have no widely recognized local equivalents, making health messages difficult to understand and reducing their effectiveness. Several livestock keepers explained that they were unfamiliar with these disease names and did not fully understand the rationale behind recommended preventive measures.

Community actors involved in community-based surveillance reported receiving basic training on disease notification for selected priority zoonoses, including viral haemorrhagic fevers such as CCHF. However, none had received training in mental health or psychosocial support, including psychological first aid. They also described limited involvement in surveillance planning and decision-making, with their participation largely restricted to outbreak response. Respondents further highlighted inadequate institutional recognition, insufficient financial and logistical resources, and persistent structural barriers that limited the effectiveness and sustainability of community-based surveillance activities.

## 4 Discussion

This study has examined the operationalization of the OH approach in Senegal through both systems-thinking and socio-anthropological perspectives. A systems-thinking approach conceptualizes epidemic preparedness and response as complex adaptive systems shaped by dynamic interactions between institutional, social, environmental, behavioral, and political subsystems rather than isolated biomedical events (18, 48). Complementarily, a socio-anthropological approach elicits that public responses to epidemic management are deeply influenced by local beliefs, social norms, trust relations, lived experiences, and historical interactions between communities and health institutions (19–21). Through these rwo approaches, the findings show that although OH has become increasingly institutionalized, its implementation remains fragmented,and insufficiently integrated with psychosocial and community realities (6) The findings reveal important feedback dynamics between institutional practices and community perceptions. Limited community engagement and poorly adapted communication strategies have contributed to mistrust and reduced adherence to public health measures, which in turn complicated outbreak control efforts and reinforced institutional reliance on top-down interventions. This cyclical dynamic illustrates how exclusion of community perspectives may unintentionally weaken the effectiveness of preparedness systems themselves.

### 4.1 Mental health as a neglected dimension of OH preparedness and response

The findings show that in Senegal the OH approach remains largely biomedical in practice, with multisectoral coordination focused primarily on human and veterinary health. Mental health is notably absent from zoonotic disease surveillance and preparedness discussions, reflecting a narrow conceptualization of health within OH governance. Frameworks that overlook psychosocial wellbeing fail to address the full scope of health impacts during zoonotic crises (6, 8).

The current operationalization of OH remains dominated by technical and epidemiological priorities. From a systems-thinking perspective, this cannot be reduced to disease detection and biomedical control measures alone, as health outcomes emerge from dynamic interactions between social, institutional, cultural, psychological, and environmental systems (18, 48). Although institutional mechanisms such as COUS and EMIS-Psy exist, mental health interventions in the Senegalses setting are treated as peripheral or crisis-specific rather than as integral components of preparedness systems. Consistent with prior studies, mental health continues to be neglected in epidemic responses, particularly in low-resource settings (11, 22).

The limited participation of psychologists, psychiatrists, socio-anthropologists, and social workers in OH coordination further reflects institutional hierarchies and disciplinary silos. In this study, unclear institutional roles, weak interdisciplinary collaboration, and insufficient OH training further constrained the contribution of mental health professionals to multisectoral planning and response activities. Internal coordination weaknesses within the mental health sector further reduce its visibility and influence. Similar dynamics have been documented in African health systems, where mental health services frequently occupy a marginal position due to limited funding, low political prioritization, and persistent sociocultural stigma surrounding mental illness (23).

During outbreaks, psychosocial support services were largely absent, while psychosocial disruptions were clear. Health workers often lacked training in trauma-informed care and empathetic communication. and social workers remained confined to administrative tasks with limited involvement in emergency response systems. Social workers’ competencies in psychological support, empathetic communication, and active listening remain underutilized and poorly recognized. Only two out of 31 patients reported receiving psychosocial support, and no formal mechanisms for this support were in place for rabies, CCHF, or post-Covid-19 outbreaks. Similar gaps were observed during Ebola and Covid-19 responses in several African countries, where psychological distress, stigma, grief, and fear significantly affected both communities and healthcare workers but remained insufficiently addressed (9, 10).

At the same time, the findings highlight the potential added value of integrating social sciences and mental health into OH systems. During the Covid-19 pandemic, spontaneous collaborations emerged in many settings between epidemiologists, psychologists, psychiatrists, and socio-anthropologists. These experiences demonstrated how interdisciplinary collaboration can strengthen risk communication, community trust, and contextual adaptation of interventions (19–21).

Community coping mechanisms relied heavily on family solidarity, religion, and traditional healing practices. While these strategies often provided emotional support and social cohesion, they also could delay seeking healthcare and reinforce stigma surrounding disease and mental distress. Integrating mental health professionals, social workers, and socio-anthropologists into preparedness and response mechanisms can improve health communication, reduce mistrust, and support more sustainable behavioural change during epidemics.

### 4.2 Fragmented operationalization of OH across institutional and community levels

The study highlights important discrepancies between the conceptual ambitions of OH and its practical implementation across governance levels. Findings revealed a fragmented OH operationalization across central and operational levels, with limited alignment between stakeholders and community realities. Although OH is increasingly supported through national coordination platforms and strategic frameworks, its operationalization remains fragmented by institutional silos, unequal power relations, and limited interdisciplinary integration. Similar implementation challenges have been documented across Sub-Saharan Africa, where OH often remains more visible in policy discourse than in routine operational practice (6, 24).

These findings illustrate how weak interactions between institutional subsystems undermine the collective functioning of preparedness systems. Coordination mechanisms frequently prioritized sectoral mandates, administrative responsibilities, and resource allocation rather than integrated problem-solving. The dominant role of the Ministry of Health, largely reinforced by external funding mechanisms and donor priorities, contributed to asymmetrical relationships between sectors, particularly with animal health, environmental, and social sectors. These findings align with previous studies showing that OH governance is often shaped by political and institutional power dynamics that weaken intersectoral collaboration in Mexico (25) and other settings (26). Challenges in data sharing across sectors and with foreign partners further undermine effective coordination.

At the operational level, understanding and implementation of OH varied substantially across professional groups. While some district medical officers and veterinarians had received OH-related training, many frontline actors, including auxiliary staff and social workers, remained unfamiliar with the approach. Coordination is often reactive, triggered by outbreaks, with poorly defined roles between sectors. This reflects broader patterns in Sub-Saharan Africa, where OH remains more a strategic discourse than a daily practice (24, 27). These findings support critiques suggesting that OH implementation often remains centered on technical interventions while insufficiently integrating social scientists and communities (28).

The findings also illustrate tensions between top-down global health strategies and local realities. Communities frequently perceived interventions as externally imposed, particularly during vaccination campaigns and restrictive control measures. Such mistrust reflects broader socio-anthropological critiques of epidemic governance emphasizing that public health interventions may fail when they overlook local social dynamics, historical experiences, and community perceptions (49, 50). Participatory epidemiology and community engagement approaches therefore offer important opportunities to strengthen OH implementation by integrating experiential knowledge and fostering local ownership (29, 30). To move from concept to practice, OH must adopt context-sensitive, field-based approaches that align stakeholder priorities with local realities and ensure donor support is adapted to national systems.

### 4.3 Community knowledge, perceptions, and implications for intervention design

The findings reveal substantial gaps between OH strategies and community knowledge, perceptions, and lived realities, particularly in rural and pastoral settings. Although communities often recognized that diseases could be transmitted between animals and humans, the concept of zoonotic diseases remained poorly understood, especially for emerging diseases such as CCHF. Low knowledge levels were significantly associated with limited formal education, consistent with previous studies conducted in African pastoral settings (24).

Communication barriers further constrained community engagement. Public health messaging was frequently delivered in dominant national languages such as French or Wolof, excluding populations speaking other local languages, particularly Fulani in the researched pastoral communities. These language mismatches hampered risk communication effectiveness and reinforced distrust between health institutions and communities. From a socio-anthropological perspective, language is not merely a communication tool, but also shapes trust, inclusion, and access to knowledge within health systems. Gender dynamics also influenced knowledge dissemination and participation in decision-making processes. In pastoral communities, patriarchal social structures can strictly limit women’s participation despite their central role in caregiving and livestock management. Women in this research were often excluded from interviews and medical decision processes, highlighting the need for gender-sensitive approaches in OH interventions (31).

Several community practices, including close human-animal cohabitation, collective living arrangements, and reliance on traditional explanatory models of illness, sometimes conflicted with biomedical recommendations. Tensions emerged particularly when restrictive interventions such as isolation measures or safe burial protocols were implemented without adequate community dialogue. These findings are consistent with previous Ebola studies demonstrating that public health interventions are more likely to fail when local cultural norms and social realities are insufficiently considered (21, 34).

Our findings suggest the importance of integrative approaches combining scientific evidence with community experiences and local disease understandings. Participatory and community-based approaches can strengthen risk communication, improve intervention acceptability, and foster sustainable ownership of OH interventions (7, 32, 35, 36). Bridging the gap between central strategies and local realities also requires better balanced interdisciplinary collaboration. These findings support calls for culturally grounded and community-sensitive mental health interventions as aprt ofthe existing OH interventions. Community knowledge and interdisplinary collaboration should therefore be considered an essential component of epidemic preparedness systems.

This study is, to our knowledge, among the first to examine the operationalization of the OH approach in Senegal by integrating systems-thinking and socio-anthropological perspectives with a specific focus on mental health and community engagement. The mixed-methods design has enabled triangulation of multiple data sources and provided a comprehensive understanding of OH implementation across institutional and community levels.

Nevertheless, several limitations should be acknowledged. First, the qualitative sample was purposively selected and the quantitative survey was conducted in selected regions with a relatively modest sample size, limiting the generalizability of the findings. Second, qualitative data relied on participants’ self-reported experiences and perceptions, which may be subject to recall and social desirability bias. Third, logistical constraints, language diversity, and the need for translation during some interviews may have influenced the depth and interpretation of participants’ narratives. Finally, although the BRAT provided useful guidance for exploring grief-related experiences, it required contextual adaptation and was complemented by semi-structured interviews because it has not been specifically validated for epidemic-related bereavement in Senegal. Consequently, the findings should be interpreted within their specific contextual setting.

Future studies should evaluate the effectiveness of integrating mental health professionals, socio-anthropologists, and community actors into OH preparedness and response systems. Longitudinal studies are also needed to examine the long-term psychosocial consequences of epidemics and to assess whether community centered OH interventions improve trust, surveillance, and health outcomes. Economic evaluations and implementation research would further support the development of sustainable OH governance models in Senegal and other African countries (see Table 2).

**Table 2:** Design interventions through community lens.

Challenges	Community-based Interventions
Community knowledge ignored; no feedback from surveillance → Local disease knowledge (LDK) undervalued; communities only seen as information providers.	• Document and integrate LDK through participatory epidemiology. • Regularly share surveillance results with communities via radio, market meetings, and village forums. • Provide feedback on reported cases to strengthen trust, ownership, and early reporting. • Use community facilitators and local associations to co-analyze surveillance findings.
Community volunteer burnout; lack of incentives; fragile, unsustained interventions → Burden without recognition weakens engagement.	• Offer consistent micro-incentives (transport stipends, airtime, training opportunities). • Link volunteers to income-generating activities (e.g., soap/mask production, poultry farming). • Provide formal recognition through certificates, badges, or public acknowledgment during community events. • Encourage peer-mentorship between experienced and new volunteers.
Mental health and psychosocial dimensions neglected in OH preparedness and response → Stakeholders and implementers underestimate psychosocial distress; mental health actors rarely included in planning or field operations.	• Re-conceptualize stakeholder engagement in OH to *institutionally include mental health specialists* as done with social scientists during the Ebola response.• Map existing mental health resources and integrate them into preparedness and response plans.• Train human, animal, and environmental health professionals in communication, stigma reduction, trauma-informed care, and psychological first aid.• Deploy mobile MHPSS teams and community-based healing groups.• Conduct reflective sessions linking unaddressed psychosocial outcomes from Ebola and COVID-19 to current OH gaps.• Develop culturally grounded peer-support models using local healing norms and traditional mediators.
Confusion over institutional roles; distrust of external actors → Unclear referral pathways and low trust in outsiders.	• Conduct joint outreach visits by human, animal, and mental health teams. • Display posters/maps locally to clarify referral pathways (health post, veterinary post, psychosocial focal point).• Engage traditional healers, religious leaders, and socio-anthropologists to bridge cultural gaps and enhance legitimacy.
Communities excluded from planning and decision-making.	• Organize co-design workshops for preparedness and health campaigns, ensuring inclusion of women, youth, and marginalized groups. • Establish inclusive local OH committees integrating mental health and psychosocial focal points. • Use participatory tools (mobile, oral, or visual) for ongoing community monitoring and feedback.
Different knowledge and perspectives among actors; unclear roles; language barriers → Misalignment across levels and lack of dialogue.	• Translate and operationalize the OH concept for clarity across sectors.• Provide OH training at all levels (central, operational, community) with role-specific and cross-disciplinary modules, including psychology.• Use multilingual local communication (radio, community theatre, visual aids) in Pulaar, Wolof, and other relevant languages.• Train health workers, religious leaders, livestock agents, and teachers as OH and MHPSS ambassadors.• Apply oral storytelling and visual tools where literacy is low to bridge central–operational–community dialogue.

The proposed interventions highlight the need to move OH from a predominantly institutional framework toward a community-centered model of epidemic preparedness. Institutionalizing mental health and psychosocial support within OH governance, strengthening interdisciplinary collaboration, improving multilingual risk communication, and integrating community actors into preparedness planning could enhance the effectiveness, acceptability, and sustainability of epidemic response strategies.

## 5 Conclusion

This study provides new evidence on the operationalization of the OH approach in Senegal through systems-thinking and socio-anthropological perspectives. The findings may inform OH policy development in Senegal and other low- and middle-income countries facing similar governance and coordination challenges. Although OH has gained substantial institutional and political support, its implementation remains fragmented and predominantly biomedical, with limited integration of psychosocial, behavioral, and community dimensions. The study demonstrates that community knowledge and lived experiences are valuable yet underutilized resources for epidemic preparedness, while persistent gaps in communication, interdisciplinary collaboration, and community engagement continue to undermine trust and the effectiveness of public health interventions. The participatory qualitative approach was instrumental in identifying these implementation gaps, highlighting the importance of community perspectives that are often overlooked by conventional surveillance systems. Strengthening OH implementation therefore requires moving beyond sectoral coordination toward genuinely integrated, community-centered preparedness systems. Future efforts should institutionalize community participation, integrate mental health and psychosocial support into OH governance, strengthen multilingual and culturally sensitive communication and feedback mechanisms, recognize local knowledge, and promote equitable interdisciplinary collaboration. By adopting a more holistic and socially grounded approach, OH can better respond to the complex realities of emerging infectious diseases while strengthening health system resilience in Senegal and comparable settings (see Figure 9).

**Figure 9:**
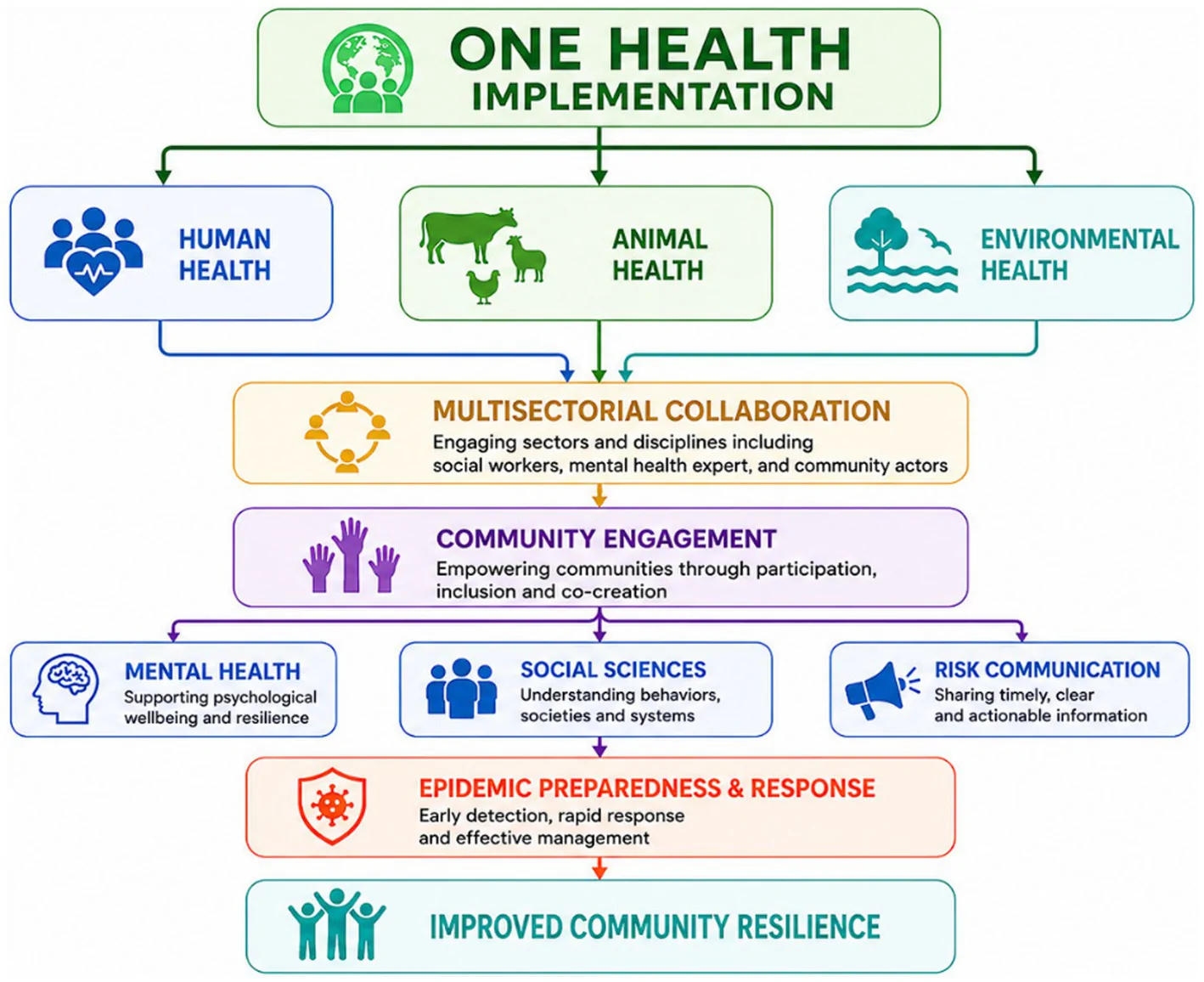
OH coordination framework of the study.

## Data Availability

The original contributions presented in the study are included in the article/Supplementary material, further inquiries can be directed to the corresponding author.
